# Supercritical CO_2_ Curing of Resource-Recycling Secondary Cement Products Containing Concrete Sludge Waste as Main Materials

**DOI:** 10.3390/ma15134581

**Published:** 2022-06-29

**Authors:** Min-Sung Kim, Sang-Rak Sim, Dong-Woo Ryu

**Affiliations:** Department of Architectural Engineering, Daejin University, Gyeonggi-do 11159, Korea; jklm2@naver.com (M.-S.K.); simsr@daejin.ac.kr (S.-R.S.)

**Keywords:** concrete sludge waste, supercritical CO_2_ curing, secondary cement product

## Abstract

This study aims to develop highly durable, mineral carbonation-based, resource-recycling, secondary cement products based on supercritical carbon dioxide (CO_2_) curing as part of carbon capture utilization technology that permanently fixes captured CO_2_. To investigate the basic characteristics of secondary cement products containing concrete sludge waste (CSW) as the main materials after supercritical CO_2_ curing, the compressive strengths of the paste and mortar (fabricated by using CSW as the main binder), ordinary Portland cement, blast furnace slag powder, and fly ash as admixtures were evaluated to derive the optimal mixture for secondary products. The carbonation curing method that can promote the surface densification (intensive CaCO_3_ formation) of the hardened body within a short period of time using supercritical CO_2_ curing was defined as “Lean Carbonation”. The optimal curing conditions were derived by evaluating the compressive strength and durability improvement effects of applying Lean Carbonation to secondary product specimens. As a result of the experiment, for specimens subjected to Lean Carbonation, compressive strength increased by up to 12%, and the carbonation penetration resistance also increased by more than 50%. The optimal conditions for Lean Carbonation used to improve compressive strength and durability were found to be 35 °C, 80 bar, and 1 min.

## 1. Introduction

The demand for concrete is increasing considerably, owing to industrial development and population growth. For cement, the core material of concrete, CO_2_ emissions attributed to the decarboxylation (CaCO_3_ → CaO + CO_2_) of limestone (the main raw material) during the high-temperature firing process at ≥1450 °C account for approximately 60% of the total, and CO_2_ emissions owing to the combustion of fossil fuel during equipment operations account for the remaining 40% [1,2]. CO_2_ emissions based on the cement firing process represent approximately 8% of the global CO_2_ emissions [3]. Thus, the cement industry is classified as an industry associated with considerable carbon emissions [4].

In recent years, various efforts have been expended to reduce greenhouse gas (GHG) emissions worldwide to prevent sudden changes in climate owing to global warming. However, carbon emissions in South Korea are increasing every year. The 2030 GHG reduction target was set to 37% compared with the Business-As-Usual at the time of the Paris Climate Agreement but was recently increased to 40% compared with emissions in 2018, based on considerations of the international GHG reduction trend [5]. Despite this policy change, CO_2_ emissions from the cement industry are increasing every year. As a result, there is an urgent requirement to develop carbon capture utilization (CCU) technology that utilizes captured CO_2_ as a resource through permanent fixation to realize carbon neutrality in accordance with the current global trend.

In general, the carbonation of concrete causes the corrosion of steel reinforcement by lowering the alkalinity of the concrete cover as shown in Equation (1), and in the long-term, as represented in Equation (2), carbonation causes decomposition of C–S–H structures, thus degrading the concrete durability [6].

Ca(OH)_2_ + CO_2_ → CaCO_3_ + H_2_O (1)

(CaO)_x_(SiO_2_)(H_2_O) + xCO_2_ → xCaCO_3_ + SiO_2_(H_2_O)t + (z − t)H_2_O (2)

However, in a previous study of Groves et al. [7], experiments on cement paste carbonation were conducted using pastes of C_3_S to examine and measure the rate of carbonation reaction of portlandite and C–S–H, which are the main hydration products of a cement-hardened body. As a result, it was reported that in the initial stage of carbonation reaction, portlandite showed a fast reaction rate but over time, microcrystalline CaCO_3_ layers of relatively high density were formed on the surface of the portlandite crystals, leading to a decrease in reaction rate, and after the completion of the reaction of portlandite, the carbonation reaction rate of C–S–H increased. Houst and Wittmann [8] reported that the carbonized Portland cement paste samples showed a decrease in water absorption rate compared to that of the non-carbonated samples.

As discussed above, recent studies have reported that CaCO_3_ formed by the reaction between Ca(OH)_2_ and CO_2_ is expected to improve the mechanical properties and durability by filling the pores in concrete [9,10,11,12,13]. Considering that the carbonation reactivity of typical CO_2_ is low, subject to the typical atmospheric temperature and pressure conditions, the studies using supercritical CO_2_ above the critical point (31.1 °C and 73.8 bar) to maximize the carbonation reaction have recently increased [14,15,16,17,18].

Meanwhile, the shipment of ready-mixed concrete is also increasing every year, owing to the increased demand for concrete, with concrete slurry water inevitably generated during the concrete production process. Considering that concrete slurry water is composed of supernatant water and concrete sludge waste (CSW), containing a large amount of unhydrated cement particles, the research efforts expended to recycle CSW as a cementitious material have been increasing during the past few years [19,20,21,22]. The results of previous studies on the effective use of CSW can be mainly divided into dry and dehydrated CSW depending on the pretreatment method. For dry CSW, the strength and durability are lowered by the needle-type ettringite formed by the accelerated hydration reaction of unhydrated cement particles owing to the high-temperature drying process [23,24]. Conversely, in the case of dehydrated CSW, using dehydrated CSW immediately after the collection of concrete slurry water is appropriate because additional hydration reactions cannot be expected, with the moisture content increasing, owing to the hydration of unhydrated cement particles as the retention period in concrete slurry water increases [25,26]. Therefore, for CSW dehydrated immediately after collection, the development of strength is possible, owing to unhydrated cement particles, and improvements in mechanical properties, as well as CO_2_ fixation, can be expected to be achieved by carbonation curing [27,28].

**Figure 1:**
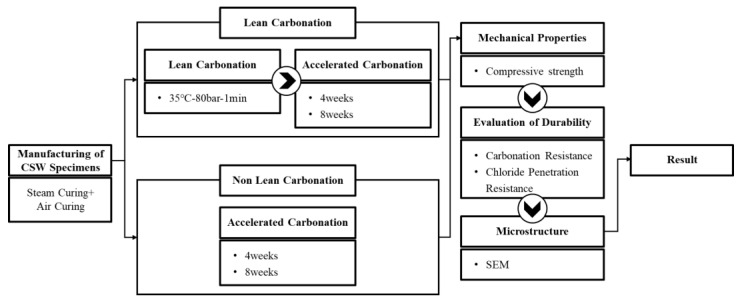
Research flow of this study.

As such, in this study, the reaction that can promote the surface densification of the hardened body within a short period of time using supercritical CO_2_ curing was defined as “Lean Carbonation”, and the mechanical properties of mortar that used CSW as the main material, as well as the durability improvement effect (carbonation and chloride penetration resistance) of Lean Carbonation, were examined for the development of highly durable, resource-recycling secondary cement products. Figure 1 shows the research flow of this study.

## 2. Derivation of the Optimal Mixture for Resource-recycling of Secondary Cement Products

### 2.1. Experimental Plan and Method

#### 2.1.1. Analysis of CSW Moisture Content Based on Pressurized Dehydration

**Figure 2:**
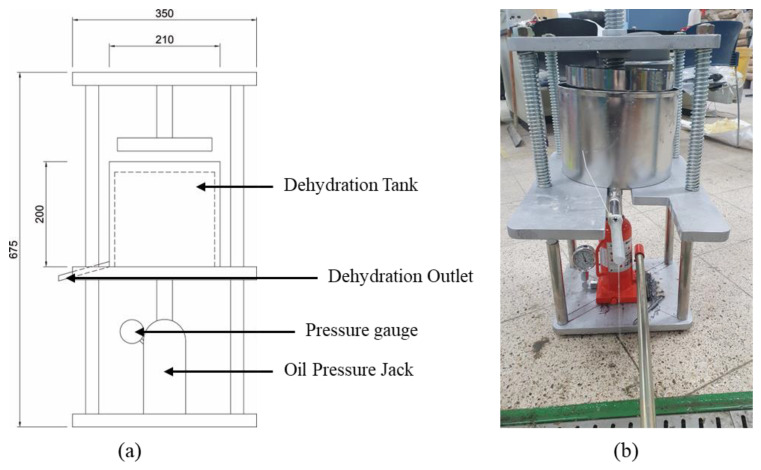
Pressurized dehydration equipment: (**a**) conceptual diagram and (**b**) installation example.

In this study, pressurized dehydration equipment, identical to that used in the filter press method, was fabricated to use dehydrated CSW, as shown in Figure 2, with a pressure gauge installed to quantify the applied pressure.

Before the main experiment, the optimal pressurized dehydration conditions (400 bar, 5 min, and 3 times) were derived based on a preliminary experiment. The moisture content tendency of dehydrated CSW was evaluated according to the retention period in concrete slurry water.

#### 2.1.2. Materials Used and Mixture Proportions

The CSW used in this study was collected from the ready-mixed concrete factory of company Y located in Gyeonggi-do, Korea, and was produced on the same day. Recovered sand was used as fine aggregate and supernatant water as mixing water. Table 1 and Table 2 list the chemical compositions of supernatant water and CSW.

**Figure 3:**
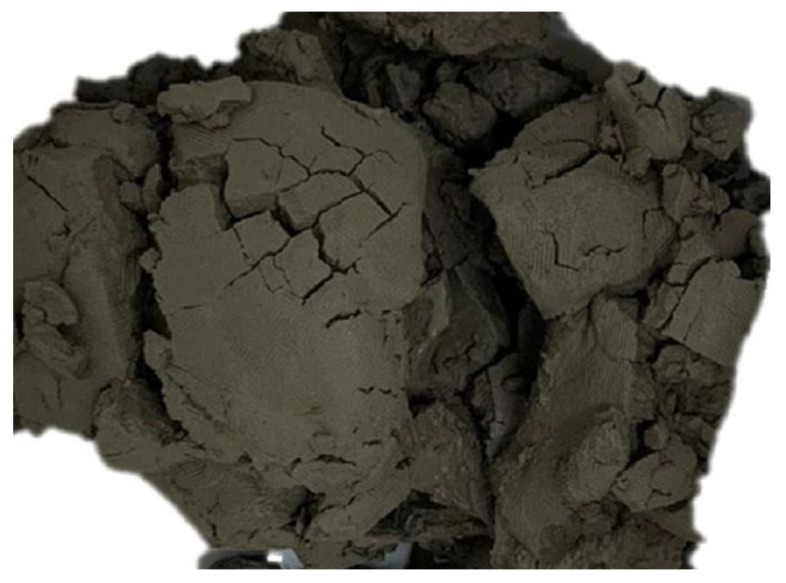
Concrete sludge waste (CSW) immediately after dehydration with moisture content of 40%.

To derive the optimal mixture (mix) for resource-recycling secondary cement products, the CSW subjected to pressurized dehydration immediately after collection (moisture content: 40%, specific gravity: 2.69) was used as the main binder; ordinary Portland cement (OPC), blast furnace slag (BS) powder, and fly ash (FA) were added as admixtures based on the CSW weight in the experiment. As for the dehydration process of CSW, Figure 3 shows the image of CSW immediately after dehydration. To secure fluidity as the property of unhardened mortar, mortar mixing was performed by setting the slump flow of all mixtures to be equal to 150 ± 15 mm. The compressive strengths of resource-recycling paste and mortar were evaluated to derive the optimal mix proportions. The experimental factors and levels are listed in Table 3, and the mix proportions of paste are listed in Table 4. In the case of mortar, the ratios of binder and sand were set to 1:1, 1:2, and 1:3, with the mixtures named Mortar 1:1, Mortar 1:2, and Mortar 1:3, respectively and the mix proportions of mortar are listed in Table 5, Table 6 and Table 7.

#### 2.1.3. Compressive Strength

**Figure 4:**
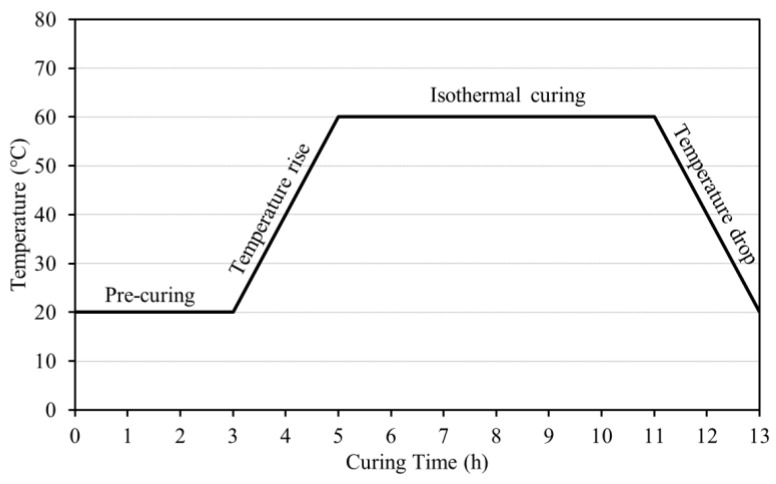
Steam curing method.

For compressive strength tests and measurements, cubic specimens were prepared (50 × 50 × 50 mm^3^) in accordance with the KS L 5105 standard (compressive strength test method for hydraulic cement mortar). Considering the curing method, steam curing was performed at an accumulated temperature of 500 °C·h with pre-curing (20 °C and 3 h), temperature rise (20 °C/h and 2 h), and isothermal curing (60 °C and 6 h) settings in accordance with KS L 4004 (concrete block), as shown in Figure 4. Compressive strength was measured for specimens subjected to a climatic chamber at constant temperature and relative humidity (20 °C, 60% RH) after steam curing; for all mixtures, the average value of the compressive strength of the three tested specimens was measured to determine the compressive strength.

### 2.2. Experiment Results and Analysis

#### 2.2.1. CSW Moisture Content Measurement Results Based on Pressurized Dehydration

**Figure 5:**
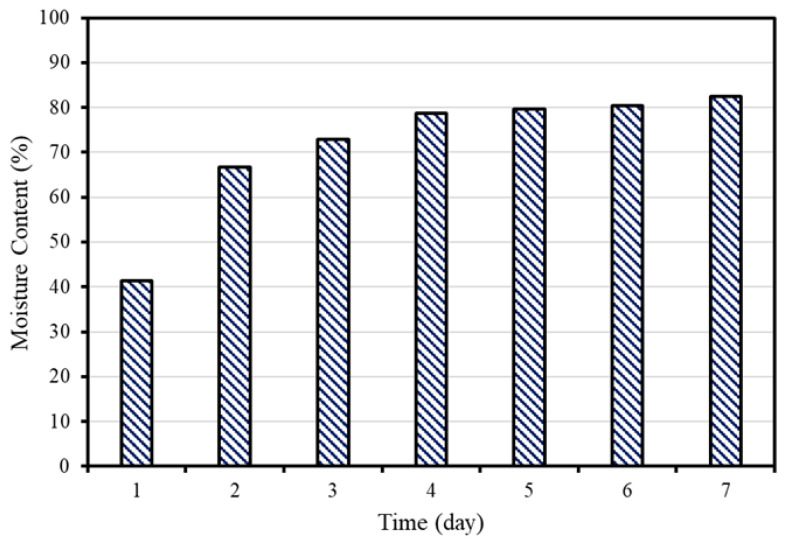
Moisture content of concrete sludge waste (CSW) according to the retention period.

Figure 5 shows the moisture content according to the retention period in concrete slurry water. Immediately after collection, the moisture content of CSW was measured to be approximately equal to 40%. The moisture content increased to approximately 67% after 1 d and to 82% after 6 d, confirming that the moisture content rapidly increased at the beginning but gradually converged to a value as the retention period increased, consistent with the results of previous studies in which the moisture content increased alongside the increase in the hydration products of cement [23,24,25]. Therefore, in the case of CSW used for resource-recycling secondary cement products, it is deemed appropriate to dehydrate and use the CSW collected on the same day because it is rich in unhydrated cement particles.

#### 2.2.2. Compressive Strength Measurement Results

**Figure 6:**
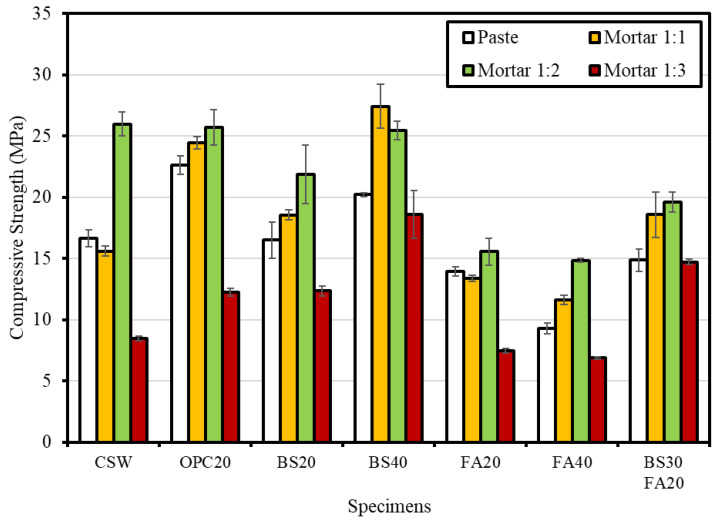
Compressive strength measurement results.

Figure 6 shows the compressive strength measurement results. Regardless of the type and content of admixture, Mortar 1:2 exhibited the highest compressive strength (21 MPa) on average followed by Mortar 1:1 (18 MPa), paste (16 MPa), and Mortar 1:3 (11 MPa).

Considering the average compressive strength by admixture, BS40 yielded the highest value (22.9 MPa) followed by OPC20 (21.2 MPa), BS20 (17.3 MPa), BS30FA20 (16.9 MPa), CSW (16.7 MPa), FA20 (12.6 MPa), and FA40 (10.7 MPa). In the case of the mixture with CSW alone, compressive strength was measured to be lower than the mixture containing BS powder or OPC as admixtures, but the strength was higher than the mixture with fly ash. The result is possibly attributed to the dehydrated CSW with a large amount of unhydrated cement particles contributing to the development of compressive strength [24]. In the case of the BS mixtures, it appears that the compressive strength was measured to be high as a result of the hydration reaction because a sufficient amount of an alkali activator [Ca(OH)_2_] was contained in the components of CSW to destroy the glass structure of BS [29]. Conversely, in the case of the FA mixtures, the lowest compressive strength occurred because the amount of Ca(OH)_2_ used for the pozzolanic reaction was not sufficient. According to Papadakis [30], FA lowers the compressive strength when its content exceeds the optimal content (25% compared with cement when the calcium content of FA is low). A similar tendency was observed in this study.

The comprehensive evaluation of compressive strength showed that the optimal mix that satisfies the KS F 4004 (concrete brick) criteria (13 MPa or higher for type 1 bricks and 8 MPa or higher for type 2 bricks) is Mortar 1:2, with the highest compressive strength. The mix that used CSW as a single binder also satisfied the compressive strength criterion for type 1 bricks in the KS standard, confirming that the compressive strength was improved when cement and BS powder were added as admixtures.

## 3. Derivation of the Optimal Conditions for Lean Carbonation

### 3.1. Concept of Lean Carbonation

**Figure 7:**
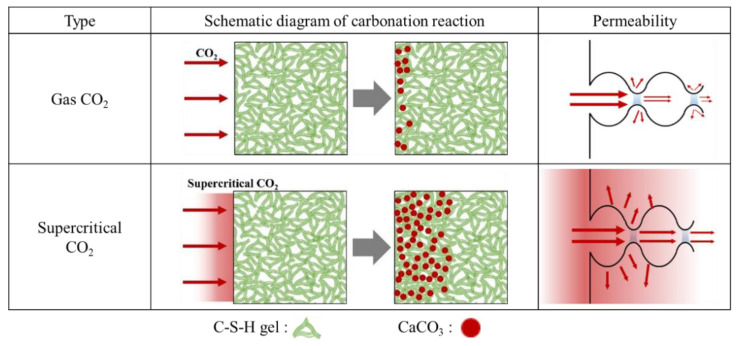
Comparison between accelerated carbonation and supercritical CO_2_ curing.

**Figure 8:**
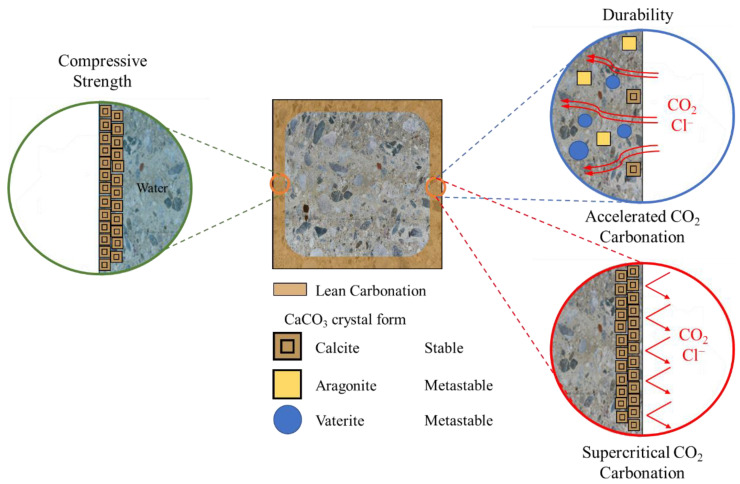
Expected effects of Lean Carbonation.

While accelerated carbonation requires a long reaction time—owing to the diffusion-controlled reaction during which CO_2_ gas penetrates the pore structure, dissolves in pore water, and reacts with Ca(OH)_2_—the carbonation reaction through supercritical CO_2_ does not decrease the reaction rate owing to its high permeability, possibly increasing the carbonation reaction rate compared with gaseous CO_2_ [31]. Figure 7 shows the difference between Lean Carbonation (defined in this study) and the gaseous CO_2_ carbonation mechanism. The expected durability (carbonation and chloride penetration resistance) improvement effect of Lean Carbonation is shown in Figure 8.

### 3.2. Experimental Method Used to Derive Optimal Conditions for Lean Carbonation

#### 3.2.1. Supercritical CO_2_ Curing Equipment

**Figure 9:**
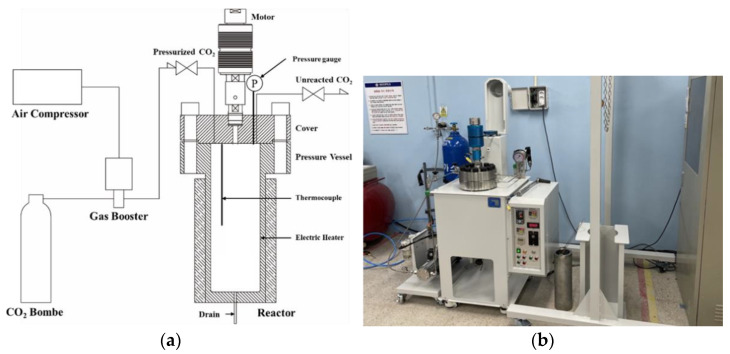
Supercritical carbon dioxide (CO_2_) reactor. (**a**) Conceptual diagram of the supercritical CO_2_ reactor; (**b**) Installation example of the supercritical CO_2_ reactor.

In this study, supercritical CO_2_ curing equipment was fabricated to perform Lean Carbonation, as shown in Figure 9. The equipment consists of a reactor (4 L) and a pressurization device, with a thermocouple and pressure gauge installed in the reactor (4 L) to control the temperature and pressure.

#### 3.2.2. Supercritical CO_2_ Curing Conditions

To derive the optimal curing conditions for Lean Carbonation, Φ100 × 100 mm^3^ specimens (Mortar 1:2 and the mix that used CSW as a single binder) were prepared in accordance with the KS F 2584 standard (test method for accelerated carbonation of concrete). Epoxy was applied to the top and bottom surfaces of the specimens for the unidirectional penetration of CO_2_ after seven days of steam curing. In the process used for quantifying the supercritical CO_2_ curing reaction time, the specimens were placed in the reactor (4 L) and the target temperature inside the reactor was reached by heating the heating plate. Pure CO_2_ gas (99.9%) was then injected under pressurized conditions through a gas booster until the target pressure was reached. During the determined reaction time, specimens were undergoing Lean Carbonation. Most studies on mineral carbonation based on supercritical CO_2_ were conducted in the ranges of 30–50 °C and 80–100 bar [32]. Based on these conditions, in this study, the temperature and pressure conditions were set to 35 °C and 80 bar and 40 °C and 100 bar, close to the supercritical CO_2_ critical point (31.1 °C and 73.8 bar); the carbonation depth was measured by varying the curing time from 1 to 5, 10, 30, and 60 min.

#### 3.2.3. Evaluation of the Optimal Conditions for Lean Carbonation

The specimens were split after supercritical CO_2_ curing, and a 1% phenolphthalein solution was sprayed onto the cross-section to derive the optimal curing conditions for Lean Carbonation. The optimal supercritical CO_2_ curing conditions were then selected by measuring the carbonation depth of the area, where discoloration did not occur.

### 3.3. Derivation of the Optimal Condition Outcomes for Lean Carbonation

**Figure 10:**
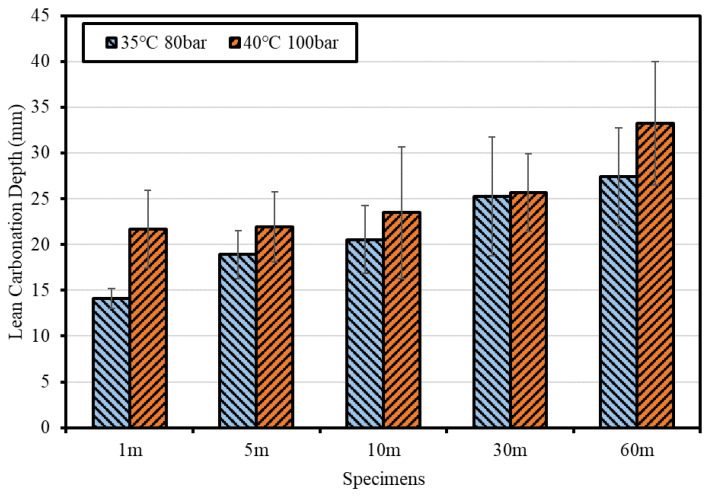
Carbonation depth measurement results after supercritical CO_2_ curing.

Figure 10 shows the carbonation depth results after supercritical CO_2_ curing. The carbonation depth increased as the temperature, pressure, and curing time increased. However, the interface was nonlinear because carbonation did not occur uniformly from the surface.

According to Rimmele et al. [33], cement paste was composed of the carbonated zone, carbonation front, and inner part of the sample in supercritical CO_2_ exposure conditions, with the densest structure formed at the carbonation front. In the case of Lean Carbonation, the optimal supercritical CO_2_ curing conditions used to improve durability by causing surface densification through the formation of CaCO_3_ were evaluated to be 35 °C, 80 bar, and 1 min.

## 4. Evaluation of Mechanical Properties and Durability Based on Lean Carbonation

### 4.1. Experimental Plan and Method for Lean Carbonation

Lean Carbonation (35 °C, 80 bar, 1 min) was performed for the specimens of CSW, OPC20, BS20, and FA20 mixtures based on Mortar 1:2, and was considered the most suitable for resource-recycling secondary cement products. The specimens were subjected to steam curing and air-dry curing for 7 days before Lean Carbonation. The Lean Carbonation depth was examined for each mixture, and compressive strength and durability (carbonation resistance and chloride penetration resistance) were compared and evaluated depending on whether Lean Carbonation was applied. The curing conditions are listed in Table 8, and the experimental factors and levels in Table 9.

#### 4.1.1. Carbonation Depth at Different Mixtures

The carbonation depth was measured in different mixtures in the same way as when the optimal conditions for Lean Carbonation were derived.

#### 4.1.2. Compressive Strength

Compressive strength specimens were prepared (50 × 50 × 50 mm^3^), and the compressive strengths before and after Lean Carbonation were compared and evaluated.

#### 4.1.3. Carbonation Resistance

**Figure 11:**
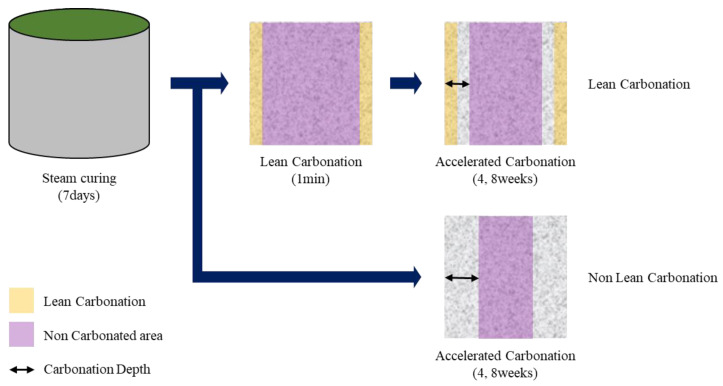
Carbonation resistance evaluation method.

Carbonation resistance was evaluated in accordance with the KS F 2584 standard (test method for accelerated carbonation of concrete) after preparing Φ100 × 100 mm^3^ specimens. The steam curing specimens were cured for 4 weeks in constant temperature and humidity conditions (20 ± 1 °C and 60 ± 1% RH) and subjected to the accelerated carbonation test (20 ± 1 °C, 60 ± 1% RH, and CO_2_ concentration of 5%) for 4 and 8 weeks. For the Lean Carbonation specimens, the accelerated carbonation test (20 ± 1 °C, 60 ± 1% RH, and CO_2_ concentration of 5%) was conducted for 4 and 8 weeks immediately after supercritical CO_2_ curing. The carbonation resistance was compared and evaluated by measuring the carbonation depth after splitting the specimens after spraying a 1% phenolphthalein solution upon the completion of the accelerated carbonation test. Figure 11 shows the evaluation method.

#### 4.1.4. Chloride Penetration Resistance

For the chloride penetration resistance, the chloride diffusion test was conducted on the specimens before and after performing Lean Carbonation based on the NT Build 492 standard, and the calculated chloride diffusion coefficients were compared and evaluated.

#### 4.1.5. Microstructural Analysis

For microstructural analysis, 5 mm specimens were collected from the interface of the carbonated zone for the specimens subjected to steam curing, Lean Carbonation, and accelerated carbonation for 4 weeks. The collected specimens were subjected to D-dry, and their microstructures were compared and evaluated by scanning electron microscopy (SEM) imaging.

### 4.2. Lean Carbonation Experimental Results and Analysis

#### 4.2.1. Lean Carbonation Depth by Mixture

**Figure 12:**
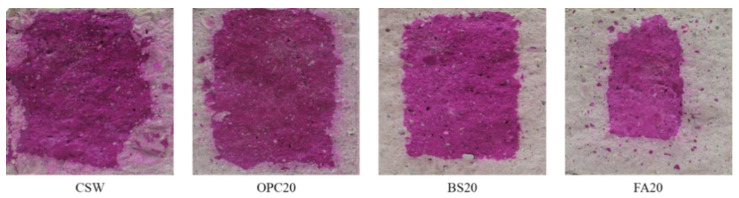
Lean Carbonation specimens.

**Figure 13:**
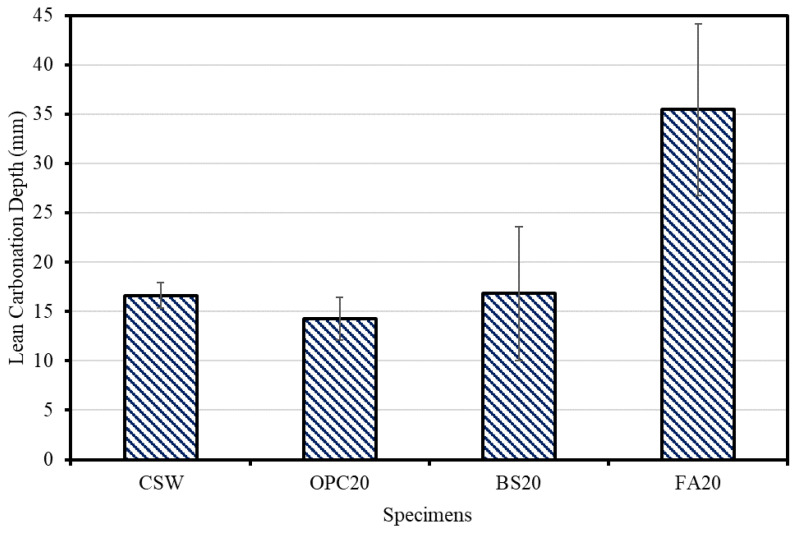
Lean Carbonation depth measurement results.

After Lean Carbonation, FA20 yielded the largest carbonation depth (35.5 mm), followed by BS20 (16.8 mm), CSW (16.6 mm), and OPC20 (14.2 mm). The FA20 mixture exhibited a significantly larger carbonation depth than the other mixtures, possibly because the carbonation resistance was lowered as Ca(OH)_2_ was rapidly consumed by the pozzolanic reaction and strength development was not sufficient [29]. Figure 12 and Figure 13 show the Lean Carbonation depth as a function of mixture.

#### 4.2.2. Compressive Strength Measurement Results

**Figure 14:**
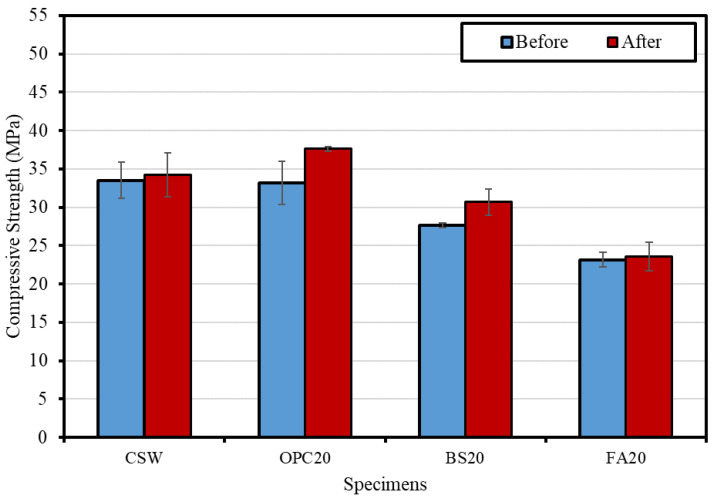
Compressive strengths before and after Lean Carbonation for different mixtures.

Figure 14 shows the compressive strength change before and after Lean Carbonation. After Lean Carbonation, the compressive strength increased for all mixtures. OPC20 exhibited the highest compressive strength increase rate (approximately 13.4%), followed by BS20 (11.0%), CSW (2.2%), and FA20 (2.0%). The compressive strength appears to have increased because the pore structure inside the specimens became denser owing to Lean Carbonation [27].

#### 4.2.3. Carbonation Resistance Evaluation Results

**Figure 15:**
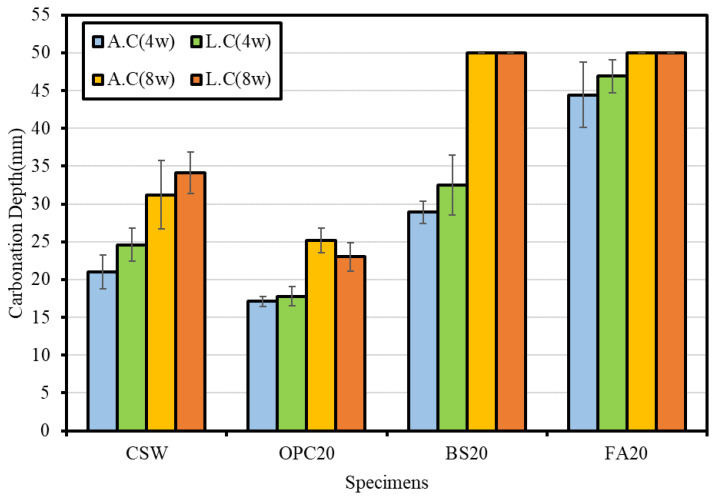
Carbonation depth measurement results.

**Figure 16:**
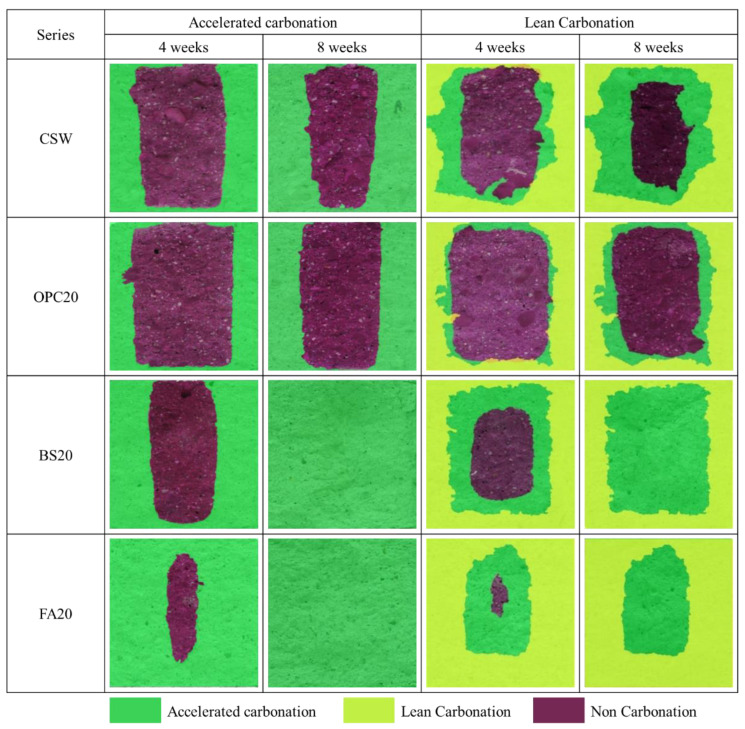
Carbonation depth measurement results before and after Lean Carbonation.

Figure 15 and Figure 16 show the carbonation resistance evaluation results. The specimens subjected to steam curing and accelerated carbonation were marked with the symbol A.C, while those subjected to Lean Carbonation and then accelerated carbonation were marked with the symbol L.C.

When the carbonation depth was measured by conducting the accelerated carbonation test after Lean Carbonation, carbonation progressed through the epoxy coating applied to the top and bottom surfaces of the specimens, owing to the high permeability of supercritical CO_2_.

For the specimens subjected to the accelerated carbonation test after steam curing, FA20 exhibited the largest carbonation depth, followed by BS20, CSW, and OPC20. The carbonation depths were 44.4, 28.9, 21.0, and 17.1 mm after 4 weeks and 50, 50, 31.2, and 25.5 mm after 8 weeks, respectively. It appears that the mixtures containing BS powder and FA yielded relatively larger carbonation depths because their carbonation resistances decreased, owing to the temporary increase in pore volume. This was caused by incomplete hydration when Ca(OH)_2_ was consumed by latent hydraulic properties and pozzolanic reactions during the hydration reaction, but sufficient wet curing was not performed at the beginning of curing [34].

For the specimens subjected to the accelerated carbonation test after Lean Carbonation, FA20 yielded the largest carbonation depth followed by BS20, CSW, and OPC20. The carbonation depths were 46.9, 32.5, 24.6, and 17.8 mm after 4 weeks and 50, 50, 34.1, and 23 mm after 8 weeks, respectively.

After Lean Carbonation, the carbonation depth increased for most of the specimens. However, OPC20 exhibited a reduction in carbonation depth when the accelerated carbonation test was performed for 8 weeks after Lean Carbonation, compared with the case in which the accelerated carbonation was performed after steam curing. Conversely, in the case of CSW, the carbonation depth was larger when the accelerated carbonation test was conducted for 8 weeks after Lean Carbonation compared with the case in which Lean Carbonation was not performed at all. Nevertheless, the rate of change of carbonation depth from 4 to 8 weeks decreased by approximately 10%, thus indicating that the carbonation depth of a specimen subjected to Lean Carbonation is expected to decrease if carbonation curing is performed for a longer time.

#### 4.2.4. Chloride Penetration Resistance Evaluation Results

**Figure 17:**
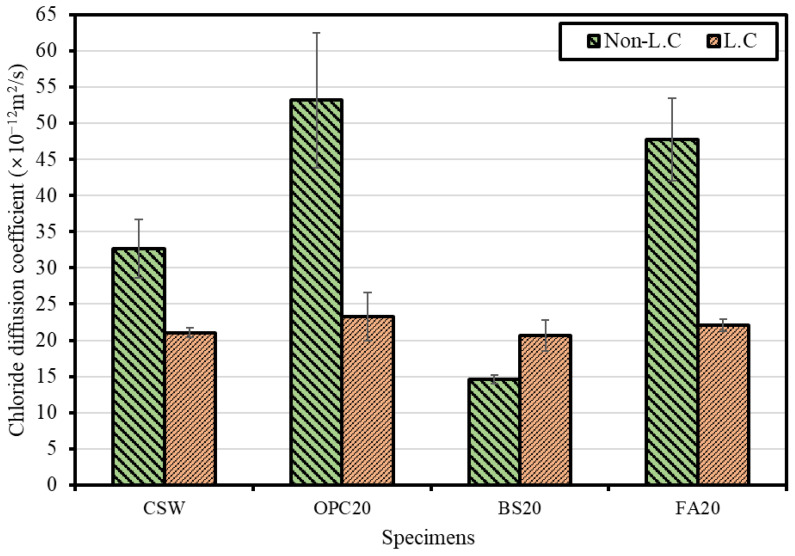
Chloride diffusion coefficient measurements.

Figure 17 shows the chloride diffusion coefficient measurement results. When Lean Carbonation was not performed, OPC20 yielded the highest chloride diffusion coefficient (53.1 × 10^−12^ m^2^/s) followed by FA20 (47.7 × 10^−12^ m^2^/s), CSW (32.7 × 10^−12^ m^2^/s), and BS20 (14.6 × 10^−12^ m^2^/s). It appears that the BS20 mix yielded the lowest chloride diffusion coefficient because it had high chloride penetration resistance as Friedel’s salt was formed through the reaction between C_3_A and chloride ions when BS powder was added [35].

When the chloride diffusion coefficient was measured after Lean Carbonation, FA20 yielded the highest value (22.1 × 10^−12^ m^2^/s) followed by OPC20 (23.3 × 10^−12^ m^2^/s), CSW (21.1 × 10^−12^ m^2^/s), and BS20 (20.7 × 10^−12^ m^2^/s). For all the mixtures (except for BS20), the chloride diffusion coefficient decreased, thus confirming the same tendency as in a previous study [36], i.e., ensuring that the diffusion of chlorides was inhibited by the densification of the pore structure caused by the formation of carbonate. However, the chloride diffusion coefficient of BS20 appeared to have increased after Lean Carbonation because the ability to fix chloride ions was lowered by the carbonation of C_3_A and monosulfate, required for the formation of Friedel’s salt. However, BS20 yielded the highest chloride penetration resistance among all mixtures despite the increase in diffusion coefficient after Lean Carbonation, possibly attributed to the densest microstructure despite the lowered ability to fix chloride ions.

#### 4.2.5. Microstructural Analysis Results

**Figure 18:**
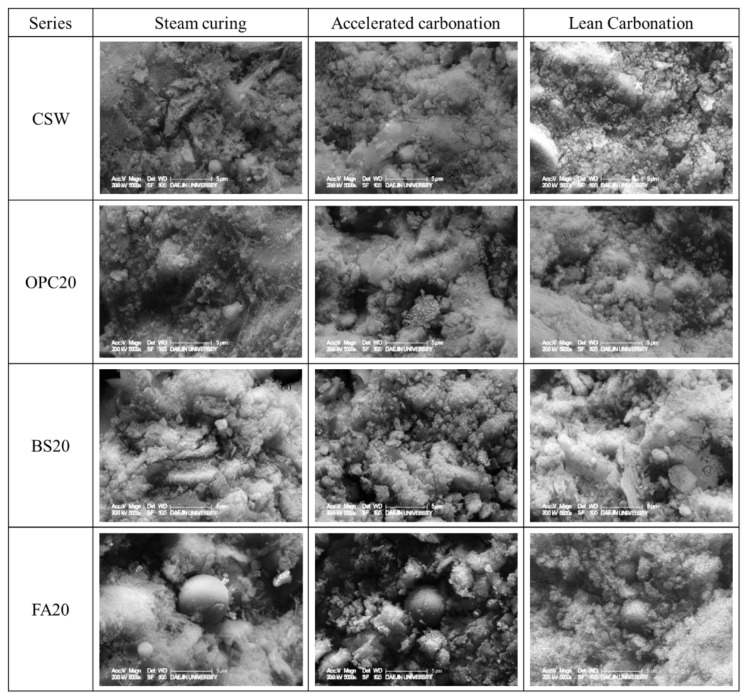
Scanning electron microscopy results (×5000).

Figure 18 lists the SEM measurement results of the various mixtures after steam curing, accelerated carbonation (4 weeks), and Lean Carbonation was performed.

**Figure 19:**
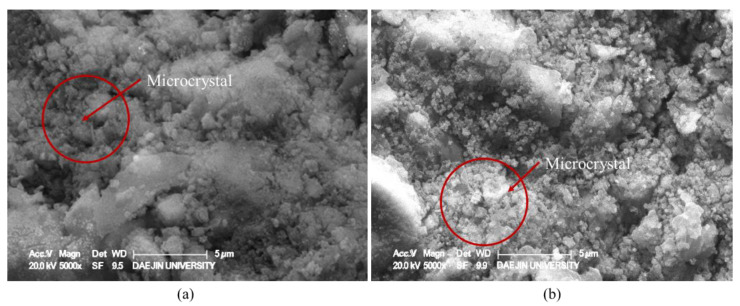
Detailed images of the formation of microcrystals inside the hardened body after the carbonation reaction (×5000). (**a**) Accelerated Carbonation and (**b**) Lean Carbonation.

In the case of the specimens subjected to accelerated carbonation and Lean Carbonation, as can be seen in Figure 19, microcrystals were generated, and the structure became denser, and in particular, more active formation of microcrystals was shown in the specimen subjected to Lean Carbonation. García-González et al. comparatively evaluated the accelerated carbonation and supercritical carbonation for OPC pastes. In their results, it was reported that the microstructure became denser, and the total pore volume decreased through the carbonation reaction. It was reported that in the case of carbonation with supercritical carbon dioxide, the effect of refinement and densification of the microstructure was observed in a few hours [35,36]. Similarly, in this study, it is considered that through accelerated and Lean Carbonation, CaCO_3_ crystals were densely formed inside the hardened body, resulting in a more dense microstructure and improved mechanical properties [37,38].

## 5. Conclusions

In this study, the optimal mixture was derived for resource-recycling secondary cement products comprising CSW from concrete slurry water as the main material. Additionally, its mechanical properties and durability were compared and evaluated depending on whether Lean Carbonation was applied for zero-emission of construction industry waste, and the realization of CCU technology was based on this condition. The experimental results are summarized as follows.

- As the retention period in concrete slurry water increased, the moisture content of CSW tended to increase. The moisture content of the CSW subjected to pressurized dehydration immediately after the collection of concrete slurry water was approximately 40%.
- Regardless of the type and content of admixture, the Mortar 1:2 mix yielded the highest strength and improved compared with the CSW mix, except for the mixtures which contained FA as an admixture.
- The compressive strength increased after Lean Carbonation, and the OPC20 mix yielded the highest strength improvement effect followed by the mixtures BS20, CSW, and FA20.
- When the carbonation resistance was evaluated by conducting the accelerated carbonation test for 8 weeks after Lean Carbonation, the carbonation depth increased, compared with the specimens not subjected to Lean Carbonation; however, the carbonation resistance increased after Lean Carbonation because the carbonation depth increase rate decreased.
- For the specimens subjected to steam curing, FA20 exhibited the highest chloride diffusion coefficient followed by OPC20, CSW, and BS20. It was confirmed that the mixture that contained BS powder had the highest chloride penetration resistance by fixing chlorides through the formation of Friedel’s salt. Meanwhile, after Lean Carbonation, the chloride diffusion coefficient decreased in all mixtures, except for BS20. It appears that chloride penetration resistance increased, owing to the dense pore structure.
- SEM analysis results revealed that the structure became denser, owing to the formation of CaCO_3_ microcrystals in micropores after accelerated and Lean Carbonation. However, in the case of Lean Carbonation, a sufficient reaction proportion occurred within shorter times compared with accelerated carbonation.

A comprehensive analysis confirmed the durability improvement effect of Lean Carbonation based on the densification of the structure. The optimal supercritical CO_2_ curing conditions were determined to be 35 °C, 80 bar, and 1 min, leading to surface carbonation.

## Figures and Tables

**Table 1:** Chemical composition of supernatant water (obtained by ICP spectroscopy).

pH	Chemical Composition (mg/L)				
	Ca	Mg	Na	Fe	K
12.5	812	0	242	0	711

**Table 2:** Chemical composition of concrete slurry waste (obtained by XRF spectroscopy).

Chemical Composition (wt.%)									
CaO	SiO_2_	Al_2_O_3_	Fe_2_O_3_	SO_3_	MgO	K_2_O	TiO_2_	Na_2_O_3_	P_2_O_5_
34.32	26.24	8.27	3.12	2.37	2.10	1.05	0.47	0.37	0.23

**Table 3:** Factors and levels.

Factors		Levels	Notes
W/B		0.4	
Specimen	CSW	100	Paste Mortar 1:1 Mortar 1:2 Mortar 1:3
	OPC	20	
	BS	20, 40	
	FA	20, 40	
	BS:FA	30:20	
Curing		Steam curing	KS L 4004
Mechanical properties		Compressive strength	KS F 5105 KS L 4004

**Table 4:** Mixture proportion—paste.

Specimens	W/B	Unit Weight (kg/m^3^)					
		W	CSW	OPC	BS	FA	Sand
CSW	0.4	518.3	1295.8	-	-	-	-
OPC20		478.9	1197.3	239.5	-	-	
BS20		475.8	1189.5	-	237.9	-	
BS40		439.7	1099.3	-	439.7	-	
FA20		464.1	1160.3	-	-	232.1	
FA40		420.2	1050.5	-	-	420.2	
BS30FA20		414.4	1036.0	-	310.8	207.2	

**Table 5:** Mixture proportion—mortar 1:1.

Specimens	W/B	Unit Weight (kg/m^3^)					
		W	CSW	OPC	BS	FA	Sand
CSW	0.4	345.5	863.7	-	-	-	863.7
OPC20		308.0	770.1	154.0	-	-	924.1
BS20		306.7	766.9	-	153.4	-	920.2
BS40		275.8	689.6	-	275.8	-	965.4
FA20		301.9	754.6	-	-	150.9	905.6
FA40		268.0	670.0	-	-	268.0	938.0
BS30FA20		259.0	647.5	-	194.2	129.5	971.2

**Table 6:** Mixture proportion—mortar 1:2.

Specimens	W/B	Unit Weight (kg/m^3^)					
		W	CSW	OPC	BS	FA	Sand
CSW	0.4	259.0	647.6	-	-	-	1295.3
OPC20		227.0	567.5	113.5	-	-	1362.1
BS20		226.3	565.8	-	113.2	-	1358.0
BS40		200.9	502.3	-	200.9	-	1406.5
FA20		223.7	559.1	-	-	111.8	1341.9
FA40		196.8	491.9	-	-	196.8	1377.3
BS30FA20		188.4	470.9	-	141.3	94.2	1412.7

**Table 7:** Mixture proportion—mortar 1:3.

Specimens	W/B	Unit Weight (kg/m^3^)					
		W	CSW	OPC	BS	FA	Sand
CSW	0.4	207.2	518.1	-	-	-	1554.4
OPC20		179.8	449.4	89.9	-	-	1617.8
BS20		179.3	448.3	-	89.7	-	1613.9
BS40		158.0	395.1	-	158.0	-	1659.2
FA20		177.6	444.1	-	-	88.8	1598.7
FA40		155.4	388.6	-	-	155.4	1632.0
BS30FA20		148.0	370.0	-	111.0	74.0	1665.0

**Table 8:** Specimen curing conditions.

Curing Method	Conditions
Steam curing	Steam curing (accumulated temperature of 500 °C·h and 11 h) → air-dry curing (20 ± 1 °C, 60 ± 1% relative humidity (RH), and up to 7 days of age)
Lean Carbonation	Steam curing (accumulated temperature of 500 °C·h and 11 h) → air-dry curing (20 ± 1 °C, 60 ± 1% RH, and up to 7 days of age) → Lean Carbonation (35 °C, 80 bar, 1 min)

**Table 9:** Experimental factors and levels.

Factors	Levels	Notes
Mix conditions	CSW, OPC, BS20, and FA20	Mortar 1:2
Curing conditions	Steam curing Lean Carbonation	Lean Carbonation (35 °C-80 bar-1 min)
Lean Carbonation depth	Carbonation depth	KS F 2584
Mechanical property	Compressive strength	KS F 5105 KS L 4004
Durability	Carbonation resistance (4 and 8 weeks)	KS F 2584
	Chloride penetration resistance	NT Build 492
Microstructure analysis	Scanning electron microscopy	Steam curing Lean Carbonation Accelerated carbonation (4 weeks)

## Data Availability

The data presented in this study are available upon request from the corresponding author.
